# The Struggles That Led to the Unwise Demise of the Public Health Laboratory Service, 1979–2003, in the Context of Changing Economic Policy

**DOI:** 10.1093/shm/hkaf049

**Published:** 2025-08-19

**Authors:** James Lancaster, Peter Roderick, Allyson M Pollock

**Affiliations:** Population Health Sciences Institute, Newcastle University, Newcastle Upon Tyne NE1 7RU, UK; Population Health Sciences Institute, Newcastle University, Newcastle Upon Tyne NE1 7RU, UK; Population Health Sciences Institute, Newcastle University, Newcastle Upon Tyne NE1 7RU, UK

**Keywords:** Public Health Laboratory Service, communicable disease control, public health history, laboratory infrastructures, civil service history

## Abstract

The Public Health Laboratory Service (PHLS) was an integral part of England’s post-war communicable disease control. Its central reference and surveillance facilities and network of laboratories in NHS hospitals was an international exemplar. Under neoliberal economic policy from the 1980s it faced intense scrutiny and expenditure constraints. Attempts to transfer its peripheral laboratories to the NHS were successfully opposed by arguing that public health needs would not be prioritised. The 1990s introduction of the internal market and capital charging fractured the complex relationships that had developed between the PHLS and NHS. Required to enter market-like contractual arrangements with NHS bodies, it found most of its funding switched from government grant to contract-generated income. This was unsustainable. The PHLS was dismantled in 2003 without a clear rationale, ostensibly because of complexity, preferential financial treatment, and professional rivalries. When Covid-19 arrived, England lacked an established and extensive public health laboratory network.

The system for communicable disease control in England was once regarded as an international exemplar. Its origins and development from the mid-nineteenth century until the start of the twenty-first century lay in a locally based and centrally supported public system, backed up by an extensive public laboratory network. Following the success of the Emergency Public Health Laboratory Service in the Second World War, its successor the Public Health Laboratory Service (PHLS), founded alongside the NHS in 1948, played a key role in what seemed like a successful battle against infectious disease. Indeed, in the 1960s and 1970s, some felt that the battle had been won. ‘The main infectious diseases which were once the major cause of death of people of working age have been virtually eliminated as health problems’ proclaimed the Department of Health and Social Security (DHSS) in 1970.^1^ Public health initiatives from then on were increasingly centred on individual lifestyles and health promotion.^2^ The public health profession and wider public engagement with public health have been central to accounts of the period.^3^

The Infected Blood Inquiry in the UK has shown how already in the 1970s and 1980s this optimistic view of infectious diseases was inadequate.^4^ Kirchhelle, in his historical account prepared for the UK Covid-19 Inquiry and elsewhere, has similarly drawn attention to structural failings and decline and their consequences.^5^

Part of the reason that the PHLS’ historical role has until recently been viewed so positively is that its histories have been written by its own employees. The standard account was written by its ex-director, Sir Robert Williams, published in 1985, with other accounts by the first two directors of PHLS’ Communicable Disease Surveillance Centre (CDSC), Spence Galbraith and Christopher Bartlett.^6^ These works all serve convincingly to advocate for the PHLS and CDSC system. PHLS’ centrality has also been shown by, for instance, Hardy in her account of salmonella, or Macfarlane and Worboys looking at the isolation of Legionnaire’s disease.^7^

However, the PHLS had many critics from at least the 1970s. It was seen as an old boys’ network, as patronising to locally based epidemiologists, and as interfering in the work of non-PHLS laboratory directors and microbiologists. Officials in the Department of Health (DH) were frustrated with its independence, which it experienced as a lack of cooperation. This was especially challenging since those officials were under strong pressure to bring down costs and to change management practices. These stemmed from neoliberal and monetarist policies that were rolled out following the election in 1979 of a Conservative government led by Margaret Thatcher until 1990 and then by John Major until 1997. They amounted to an era that saw ‘the systematic implementation of an agenda of deflation, privatisation, deregulation, and downsizing of the public sector’, and which Gorsky has described as ‘the true turning point’ for the NHS ‘with constrained expenditure, the assault on medical corporatism, the internal market, and all that has followed.’^8^ The New Labour Blair government, which was first elected in 1997, continued and extended the market-oriented and public management reforms. This was the new era of ‘governing by contract’, ill-suited to a provision of a population-level public health service.^9^

In the early 1990s, a capital charging regime was introduced, which required NHS bodies, including the PHLS, to pay from their revenue an annual charge to represent depreciation in the value of their land, buildings and equipment. This became a central mechanism of the private finance initiative, and along with other changes associated with the internal market, such as contracts and unbundling the cost of services, drove the closure of hospitals, reduction of NHS services, and growth in private provision.^10^ These developments rendered the PHLS and its network of laboratories anachronistic, and increasingly unsustainable and unworkable. Attempts were made to break it up in the 1980s, but it was not finally dismantled until 2003.

This is the first account of how civil servants and the PHLS responded to the implementation of these policies. The struggles that it recounts can be seen as an example of how cash constraints, managerial realignments and marketisation impacted more broadly on relationships between central government and arms length organisations.^11^

Williams provided a detailed historical account of the policy, structure and finance of the PHLS in 1985, and more recently, Kirchhelle has painted a thorough picture of post-war public health laboratories in the UK and elsewhere.^12^ This article presents evidence from DH and PHLS archives, including policy documents, correspondence and related papers and from a detailed consideration of PHLS financial accounts. This has enabled us to consider how changes in policy influenced the financing of an arms length public organisation (PHLS) and its relationship with central government. In this way, we draw attention to the motivations and actions of, in particular, civil servants, and provide evidence that supplements, confirms or challenges medical and scientific accounts, including the ‘selective memory’ of witness seminars (and, for that matter, popular science writing about ‘elite scientists’).^13^ These insiders can describe their own experiences and will often have a deep and valuable knowledge of their institutions but will be less well-placed to present the perspectives of others or the wider picture beyond the institution. Their statements, and those of other commentators, can also be checked against financial accounts, here explored in detail. Such a balance of government archival research, financial analysis and policy analysis we believe brings the PHLS into sharp focus in a way that may prove beneficial for other historical accounts of institutions in this period.

By describing the challenges faced by the PHLS between 1979 and 2003, we hope to contribute to an understanding of the public health infrastructure in England in the last two decades of the twentieth century, and of its place in developments in public administration in that period. While the article does not address the response in England to the Covid-19 pandemic, we believe that this account will throw some light on this and augment existing accounts such as Kirchhelle’s.

The article begins by describing the structure, development and funding of the PHLS network of central and peripheral laboratories from the 1940s to 1970s. The second section describes the civil service attempts, driven by the new policy developments and public expenditure cuts of the late 1980s, to break up the peripheral laboratory network, and shows how these attempts were successfully resisted by the PHLS. In the third section, we show the effects of the introduction of internal market policies into the NHS in the 1990s, and specifically the imposition of funding cuts coupled with the capital charging regime and contracts, on the PHLS, with reference to the relationships between the civil service and PHLS staff. In *The Road to a Communicable Disease Strategy and the Demise of PHLS* section, we consider the impact of the neoliberal policies on civil service thinking and actions, and explore the reasons for the decision in 2002 to abolish the PHLS. We consider, too, some of the repercussions of this for infectious disease control in England, with reference to the response to Covid-19.

## The Structure and Development of the PHLS

In the early twentieth century, as understanding of the bacteriological cause of infections grew, laboratories to identify pathogens were established across England by public analysts, hospitals and universities. Among those making use of these tests were medical officers of health in local authorities, who were responsible for communicable disease control in their areas. Some of these laboratories were linked to form the Emergency Public Health Laboratory Service, set up in 1938 to cover England and Wales in response to pre-war concerns about imminent biological warfare and the dangers of the spread of disease if people were evacuated from cities or if sanitary services were disrupted in bombings. A network of laboratories around a central laboratory in north London, the service was used by medical officers of health for routine work to such an extent that it was continued after the war, as the PHLS, established under section 17 of the National Health Service Act 1946, and continuing to cover England and Wales. It was run by the Medical Research Council, but following the Public Health Laboratory Service Act 1960, it was administered by the minister of health through an appointed board accountable to the minister, which was to include at least two people appointed after consultation with the Medical Research Council; at least two bacteriologists (from 1977, microbiologists); at least two medical officers of health from local authorities (from 1977 two medical officers of environmental health or the equivalent); at least one person to represent hospitals; and at least one general medical practitioner. Continued under the National Health Service Act 1977 (in order to enable it to take on in 1979 the Microbiological Research Establishment at Porton Down), it was a body corporate with executive functions that was not, according to the civil service classification of non-departmental public bodies in 1982, part of the NHS.^14^

The service consisted of the Central Public Health Laboratory (CPHL) in Colindale, north London, and a network of about 52 regional and area laboratories (the ‘peripheral’ laboratories) (see Table 3). In the 1970s and 1980s, the CPHL consisted of specialist and reference laboratories, broadly separated into the divisions of enteric pathogens, hospital infection, and microbiological reagents and quality control, the food hygiene laboratory, the virology reference library and the national collection of type cultures. Feeding into the CPHL, its peripheral laboratories provided, like the other 300 or so microbiological laboratories in hospitals in England and Wales, diagnostic and other services to the NHS and local authorities. Unlike them, though, they had an additional public health function, sending testing data to the central laboratories and (after 1977) the PHLS’ CDSC to provide a national picture of infectious disease and providing country-wide support to local authority and health authority medical officers and environmental health officers. Some of the peripheral laboratories were also reference laboratories, that is, they provided expertise and reference samples for specific pathogens, for instance, gonococcus at Bristol and mycobacterium at Cardiff. All but two of these 52 laboratories were run jointly with NHS hospitals, with staff and costs splits between the PHLS and the health authority on a collaborative basis. Taken together this formed a national public health function grafted onto the NHS.^15^

Until the early 1990s, PHLS was mainly funded through direct government grants from the Ministry of Health (from 1968 the DHSS and after 1988 the DH) and the Welsh Office or Welsh National Assembly. NHS funding comprised two budgets, centrally financed services (CFS) budgets and the hospital and community health services (HCHS) budgets. PHLS was funded from the CFS budget while NHS hospital laboratories were funded from the HCHS budget. This was a source of tension between the NHS and PHLS as the HCHS budget was uplifted annually for inflation, whereas CFS was not. A DHSS memo in January 1984 explained the problem:

*Different funding criteria apply, and these create anomalies in service provision where there is an overlap. The requirement for the PHLSB [PHLS Board]/CFS, for example, to find substantial savings is likely to create tensions where a health authority does not find itself under similar constraints and will not accept a reduction in service provision and baulks at paying more for the same level of service.*

*Funding of the HCHS is based on assessments of need nationally … and regionally … Funding of the PHLSB is unrelated and, apart from planned capital schemes and revenue consequences, done on an ad hoc incremental/decremental basis. This leads to anomalies and disagreements.* ^16^

**Fig. 1.**
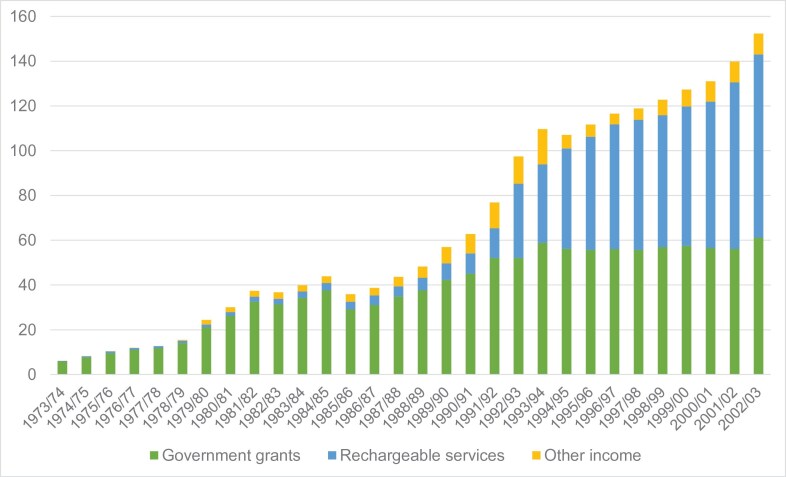
PHLS revenue income, 1973/74–2002/03, £m, cash terms

**Fig. 2.**
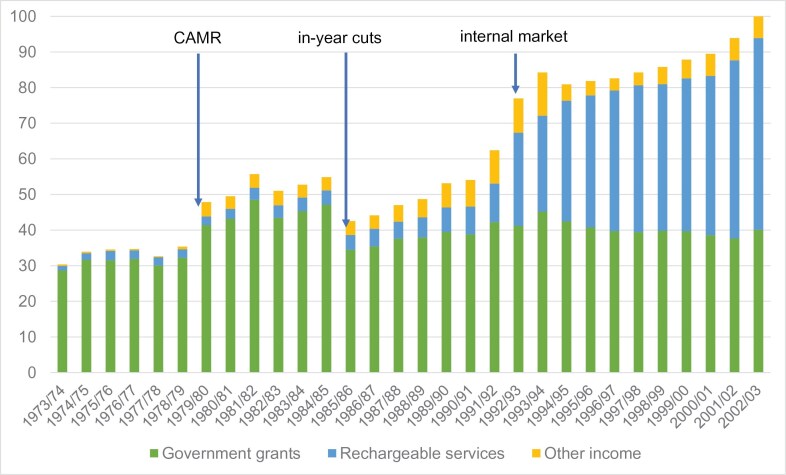
PHLS revenue income, 1973/74–2002/03, real terms, rebased to 2002/03, 2002/03 = 100

In its first year as an independent body, 1961/62, PHLS received £1m government grants (CFS), which accounted for over 97 per cent of its income. Funding increased in real terms until 1977/78. Following the UK’s 1976 International Monetary Fund crisis,^17^ the CFS grant was cut by 6 per cent in real terms and further cuts followed with a 10 per cent real terms cut from £32.6 m to £31.3 m in 1982/83 and a 27 per cent real terms cut from £37.6 m to £29.1 m in 1985/86 (Figures 1 and 2; Tables 1 and 2). (The additional funding for PHLS’ contribution to HIV/AIDS surveillance did not compensate for the real terms reductions in income.) In 1994, following a government review of all CFS spending, the permanent secretary at the DH, Sir Graham Hart, told Virginia Bottomley, secretary of state for health, that the PHLS ‘had to make a worthwhile contribution to the overall reductions in CFS’. This translated into a real terms cut for the three years after 1993/94 of 12 per cent over the period, effectively removing its inflation provision. The transfer in March 1994 of the Centre for Applied Microbiology and Research and its government grant of £5.8 m and income of £10 m out of the PHLS to the Microbiological Research Authority may have offset some of the reduction in income.

**Table 1.** PHLS revenue income, 1981/82–2002/03, £m, cash terms

	1981/82	1982/83	1983/84	1984/85	1985/86	1986/87	1987/88	1988/89	1989/90	1990/91	1991/92	1992/93	1993/94	1994/95	1995/96	1996/97	1997/98	1998/99	1999/00	2000/01	2001/02	2002/03
Government	33	31	34	38	29	31	35	38	42	45	52	52	59	56	56	56	56	57	57	57	56	61
grants (CFS)	*87%*	*85%*	*86%*	*86%*	*81%*	*80%*	*80%*	*78%*	*74%*	*72%*	*68%*	*53%*	*54%*	*52%*	*50%*	*48%*	*47%*	*46%*	*45%*	*43%*	*40%*	*40%*
Rechargeable services	2	3	3	3	3	4	4	6	7	9	13	33	35	45	51	56	58	59	62	65	74	82
	*6%*	*7%*	*7%*	*7%*	*10%*	*11%*	*10%*	*12%*	*13%*	*14%*	*17%*	*34%*	*32%*	*42%*	*45%*	*48%*	*49%*	*48%*	*49%*	*50%*	*53%*	*54%*
Other income	3	3	3	3	3	3	4	5	7	9	11	12	16	6	5	5	5	7	8	9	9	9
	*7%*	*8%*	*7%*	*7%*	*9%*	*9%*	*10%*	*10%*	*13%*	*14%*	*15%*	*13%*	*14%*	*6%*	*5%*	*4%*	*4%*	*6%*	*6%*	*7%*	*7%*	*6%*
**Total revenue income**	37	37	40	44	36	39	44	48	57	63	77	97	110	107	112	117	119	123	127	131	140	152

**Table 2.** PHLS revenue income, 1981/82–2002/03, real terms, rebased to 2002/03, 2002/03 = 100

	1981/82	1982/83	1983/84	1984/85	1985/86	1986/87	1987/88	1988/89	1989/90	1990/91	1991/92	1992/93	1993/94	1994/95	1995/96	1996/97	1997/98	1998/99	1999/00	2000/01	2001/02	2002/03
Government	48	43	45	47	34	35	38	38	39	39	42	41	45	42	41	40	39	40	40	39	38	40
grants (CFS)	*87%*	*85%*	*86%*	*86%*	*81%*	*80%*	*80%*	*78%*	*74%*	*72%*	*68%*	*53%*	*54%*	*52%*	*50%*	*48%*	*47%*	*46%*	*45%*	*43%*	*40%*	*40%*
Rechargeable services	3	3	4	4	4	5	5	6	7	8	11	26	27	34	37	39	41	41	43	45	50	54
	*6%*	*7%*	*7%*	*7%*	*10%*	*11%*	*10%*	*12%*	*13%*	*14%*	*17%*	*34%*	*32%*	*42%*	*45%*	*48%*	*49%*	*48%*	*49%*	*50%*	*53%*	*54%*
Other income	4	4	4	4	4	4	5	5	7	7	9	10	12	5	4	3	4	5	5	6	6	6
	*7%*	*8%*	*7%*	*7%*	*9%*	*9%*	*10%*	*10%*	*13%*	*14%*	*15%*	*13%*	*14%*	*6%*	*5%*	*4%*	*4%*	*6%*	*6%*	*7%*	*7%*	*6%*
**Total revenue income**	56	51	53	55	43	44	47	49	53	54	62	77	84	81	82	83	84	86	88	89	94	100

Income from capital grants, income from reserves and occasional other income are not included.

*Government grants*: funding from the Department of Health / Department of Health and Social Care from the Centrally Financed Services budget, and from the Welsh Office or Welsh National Assembly. Government capital grants transferred to revenue are not included. Consultants distinction award funding (from 1998/99) is not included. Transfers to and from reserves are not included.

*Rechargeable services*: reimbursements from the NHS and local authorities for laboratory services to cover PHLS salaries and other laboratory costs.

*Other income*: non-governmental grant funding and income from activities (sales of reagents, bench fees, etc.).

The continuous pressure on the CFS grant explains why the PHLS board continued to express the view that it was ‘underpinning the NHS to an undesirable extent’^18^ and argued for funding to switch from CFS to HCHS. As late as 1998 the PHLS was still asking that its funding be moved from CFS to HCHS because of the higher real terms growth in HCHS, and adjustment for age and demography.^19^

From 1982/83 to 1991/92 PHLS real terms income never returned to its 1981/82 level. After 1991/92 the increase in total income was in large part due to the transfer and absorption of some NHS laboratory services with the concomitant switch in income to rechargeable services from the HCHS budget. Income from rechargeable services as a proportion of all income increased from 14 per cent to 54 per cent in 1991/92 and 2001/02 respectively (see *The effect of contracting for peripheral laboratory services* section).

## Peripheral Laboratories under Pressure

Since the PHLS was funded by the DHSS to provide a service to hospitals, laboratory costs lay with the PHLS and were not, as Treasury guidance required, charged back as a service to the user. According to a DHSS finance group memorandum, ‘Costs currently lie where they fall’, so that there was ‘constant jockeying by the PHLS Board to increase its share of the resources at the health authorities’ expense.’^20^ For its part, the PHLS felt that it was spending the majority of its revenue on costs it couldn’t control and ‘to the detriment of PHLS national and community commitments’.^21^ The arrangements in place for management and cost-sharing of these laboratories had been set out in DHSS guidance HM(70)50 in 1970.

*Regional Hospital Boards should consult with the PHLS Board to ensure that, at selected Area Laboratories, the PHLS provides a microbiology laboratory as a joint (hospital/PHLS) laboratory. For hospital schemes involving joint laboratories, costs are shared on a basis related to the space provided for the PHLS and hospital purposes, agreed between the PHLS and the Board concerned. Where … the PHLS part of the laboratory is to undertake hospital bacteriology or virology the apportionment should take this into account on the basis of approximate estimates of the anticipated workload. … Arrangements for cost sharing of the revenue costs of joint laboratories should be reviewed regularly by Hospital Boards and the PHLS to ensure that they reflect broad variations in costs incurred.* ^22^

This meant that the precise arrangements varied across the laboratories, including the proportion of funding provided and the distribution of the workforce. Although it was ‘an historical “hotch-potch”’ it was at least straightforward for both parties to administer.^23^ But since the 1970s, developments in treatment and in the science and practice of routine diagnostic tests meant that the volume of tests had risen without an equivalent rise in funding, with health authorities passing the costs of treatment to the PHLS, causing it to haemorrhage resources, or at least so the board worried.^24^

These factors, combined with the PHLS’ financial struggles, led the DHSS to undertake an internal review from 1982 to 1985 to consider how costs were shared between the PHLS and district health authorities in the running of the PHLS’ 52 peripheral laboratories. The review considered that the PHLS network of laboratories allowed for good communication, gave a sense of identity (‘the family factor’), and provided good quality training. However, a centralised administration didn’t seem to bring benefits in terms of pricing or uniformity of procedures. It was expensive in staff time, and laboratories run jointly with health authorities tended to have more staff. Furthermore, ‘By maintaining its independence within the health services, PHLS microbiology enjoys a degree of protection, in as much as it does not have to compete with other medical disciplines for funds … we can see no reason why it should be treated in an even marginally privileged way.’ In particular it noted the complexity of the cost-sharing arrangements and the difficulty of disentangling them. While accepting that ‘administrative tidiness’ was not enough of a reason for structural change, the review recommended that the peripheral laboratories and their associated budgets be transferred from the PHLS to health authorities. There was no need to incur the extra costs of centrally funding and administering an independent network of laboratories that mainly provided services to health authorities, and the flow of data to the PHLS could still be guaranteed. Budgets for health authorities would be adjusted appropriately.^25^ The new organisation would look rather like the US Centers for Disease Control and Prevention.^26^

The draft review recommendations met with widespread opposition from a very wide range of professional bodies, trades unions, royal colleges, scientific, industry, and consumer bodies, government departments, local authorities, and regional medical and scientific officers.^27^ The review was seen as an attack on the PHLS. The recommendations had failed to articulate and reflect the purpose and workings of the PHLS model of centrally coordinated peripheral laboratories. In a letter to the English chief medical officer (CMO), Donald Acheson, in September 1985, the Royal College of Pathologists noted that ‘adverse comments on … the proposal that the administration and funding of peripheral laboratories should be transferred to the NHS have been features common to every submission to the College’ in response to the consultation on the review. ‘With the ever present threat of outbreaks …, with the risks of importation of exotic viral disease, and with the advent of newly recognized infections … it is important that PHLS peripheral laboratories continue to collect microbiological information of epidemiological importance and are provided with strong centrally organized support.’^28^

The two professional advisers to the review, Prof. Ian Phillips of St Thomas’ Hospital Medical School (a future member of the PHLS board) and Dr Robert Blowers, former director of PHLS Middlesbrough and chair of a PHLS quality control committee in 1970, had previously commented in October 1984 that ‘Transfer is highly undesirable … Microbiology is the only hospital service that provides information for the immediate benefit of the population as a whole and the PHLS peripheral laboratories, even if in a District Hospital, have a particular duty to do so.’ They drew attention to the important role played by ‘epidemiologically motivated microbiologists in hospital laboratories’ who were able to maintain general microbiological skills and to gain a knowledge of patterns of infection in the community at large, maintaining both patient management and epidemiological roles.^29^

Fortunately for the PHLS, it had a very active and powerful chairman of the board, Dr Charles Gordon Smith, who reported directly to the secretary of state. Appointed in 1973, he was dean of the London School of Hygiene and Tropical Medicine, had been director of the Microbiological Research Establishment at Porton Down, and while chairman was also at different times president of the Royal Society of Medicine, deputy chair of the Wellcome Trust and chair of a World Health Organization research strengthening group on tropical diseases.^30^ In August 1985, he felt able to write to secretary of state Norman Fowler about the review to complain of ‘the flimsy arguments and the lack of understanding of the problems on which this ill-founded proposal rests. Frankly, it is the Board’s view – and that of others who are well informed – that the Report totally fails to justify such a sweeping change to a major national resource.’^31^ He had separately noted that ‘NHS microbiological laboratories lack interest and impetus toward epidemiology and prevention and have prior and pressing requirement to meet clinical needs... Epidemiological work of national interest would compete poorly for funds at District level’.^32^ This would be borne out over 20 years later when laboratories were moved into the NHS.

In October 1985 Fowler, perhaps in part mindful of the PHLS’ critical role in the HIV/AIDS response, and in part responding to the PHLS board, decided not to implement any of the review’s recommendations.^33^ Civil servants felt that their actions had been misunderstood by opponents since they had been trying to save the PHLS from financial insecurity. They understood Fowler to have made a political decision, and he had made clear that discussions with the PHLS on the matter should continue.^34^ For the time being they could not see how to proceed within the government’s overall policies of marketisation, which was now the direction of travel for public services.^35^

However, although transfer to the NHS did not proceed in 1985, PHLS’ ability to retain all the peripheral laboratories was constrained. Still struggling with its costs and a possible £2 m annual deficit, the PHLS threatened to cut 300 staff. The threat was regarded by senior DHSS officials briefing John Cashman, under-secretary at the DHSS and chairman of the PHLS’ 1986 accountability review, as ‘something of an Aunt Sally and clearly untenable without real harm to the Service’.

*One interesting area to touch upon is that our crude analysis of manpower trends does not altogether support the picture of spiralling deprivation that is being presented [by PHLS]. … PHLS are puzzled by it too.* ^36^

The DHSS did consider, though, that there may be scope for some reduction in staff numbers. In a subsequent letter to the PHLS, Cashman acknowledged:

*We accept that the PHLS has managed in the recent past through a combination of short term expedience and vigorous expenditure and value for money reviews. … we are not disputing the need for, or the urgency of, a solution to your financial difficulties. As well as any deficit, the Board is arguing that an additional £1 million is needed for essential development. As we indicated, the prime source of funds for new activities must be to rethink the priority attached to old ones. …*

*The loss of 300 staff at one go without regard to functions and priorities, is clearly untenable. However, this doesn’t necessarily argue against some staff reductions.’* ^37^

Detailed plans were again drawn up in July 1986 to close another 10 laboratories. After consideration it was felt to be unwise to withdraw from all 10 laboratories but six were identified for withdrawal. In May 1987 the board heard from Malcolm Harris at the DHSS that it was not to proceed with any closures and its cash allocations for the year were increased by £2.5 m, with AIDS the first priority.^38^

## The Internal Market and Charging for Services

Further attempts by the DHSS to remedy the PHLS’ financial and organisational position now took place within the context of the plans to implement the internal market in the NHS. At this point it is worth noting that civil servants were of the view that ‘PHLS microbiology should be one of the last candidates on any list for market solutions’.^39^

However, PHLS income was already under pressure as health authorities, under financial constraints, were refusing to fund the increasing volume of diagnostic tests that clinicians and general practitioners (GPs) were referring to the PHLS laboratories, forcing the DHSS to provide extra funds to the PHLS as a temporary measure so as to protect the flow of public health data.^40^ The DHSS resurrected its proposal to shift the costs of PHLS peripheral laboratories to the NHS, this time proposing that regional health authorities (RHAs), which allocated funding to district health authorities, would contract annually with the PHLS for services within each region and meet the costs of the level of service required and any increased diagnostic work.

And so in May 1987 the NHS Management Board agreed that a proportion of 50 per cent to 60 per cent of PHLS funding for the peripheral laboratories would switch to the 14 RHAs. The PHLS would still retain a proportion of its current direct CFS grant to cover central public health functions, including work for local authorities.^41^ However, the plan to switch the budget allocations from the PHLS to RHAs could not be implemented, due to lack of management information on the costs of tests; concerns that funds for vital public health work would be subsumed into RHAs’ overall budgets and become subject to NHS financial constraints; and fears that the higher-paying RHAs would simply negotiate costs downwards to the detriment of the PHLS and other RHAs.^42^

So the fallback position was that the PHLS alone should manage public health laboratories and their staff. In 1989, the white paper *Working for patients* had proposed the introduction of the internal market into the NHS, which would be brought into effect by the NHS and Community Care Act 1990. Collaborative arrangements and unclear boundaries would no longer be possible if all parties were to be separate market players, acting as purchasers or providers. The PHLS board secretary Keith Saunders, writing in February 1990 to J Charles Dobson, the head of the DH branch dealing with the PHLS, said confidently that the board, if the sole manager of its laboratories, would ‘be able efficiently to discharge its functions in the diagnosis, surveillance and control of infectious diseases, within the framework of the Government’s White Paper’. Saunders reminded Dobson, though, that the white paper noted that ‘the public health function of the PHLS is a classic example of a service whose value to the public as a whole exceeds its value as perceived by individual customers’. Safeguards would be necessary to protect the public health, and although it would recover the costs of routine diagnostic work from RHAs, it would still require DH funding for national work, using for surveillance purposes the diagnostic data from clinical testing, carrying out food, water and environment (FW&E) analysis and surveillance for local authorities, running the central and reference laboratories and the CDSC, managing the Centre for Applied Microbiology and Research and conducting research not otherwise funded.^43^ With these caveats, the PHLS board thought that the transfer of peripheral laboratories and associated budgets and staff would provide protection *from* the market, rather than require their participation *in* the market, as understood by the DH.

In August 1990, parliamentary under-secretary of state for health, Stephen Dorrell, agreed in principle that the peripheral laboratories should be under the sole management of the PHLS.^44^ Of the 52 laboratories, 48 were now jointly run by the NHS, and by 1993 the PHLS had negotiated to take control of all 48, and around 1,000 NHS staff that worked in them were transferred to the PHLS.^45^ From the second half of 1992, the laboratories would now contract with health authorities to charge them the full cost of diagnostic tests.^46^ The PHLS (the provider) would sell diagnostic tests to NHS trusts, health authorities and GPs, as well as to local authorities (the customers or purchasers). It would also contract with the DH for various public health functions.^47^ The PHLS would now be competing to provide services with hospital laboratories and the private sector. DH funding from the CFS block grant would continue to be required for its wider public health work, but over time the majority of PHLS income would be generated from HCHS budgets in the form of rechargeable services.

### Unbundling the costs of PHLS and NHS laboratories

The transfer of NHS staff and facilities in the peripheral laboratories to PHLS was far from straightforward, as PHLS now had to unravel the longstanding arrangements with NHS bodies. A DH letter written to directors of finance in health authorities and NHS trusts in January 1993 noted that the PHLS, having received staff transferred from the NHS, was required

*to unravel the old cost-sharing arrangements and put units into a position where they know, and are funded to meet, the full costs of the level of service they have had in the past.*

*Only when this second step has been completed will units be in a position to review their service requirements and prepare contracts.*

*The task is to identify the full cost of the service provided by each PHL [public health laboratory], including a share of hospital overheads, compare the full cost with the contribution paid by the host unit in the past, and make adjustments so that each unit is funded to meet those costs from a future date [underlining in the original].* ^48^

First there was the issue of the capital charge. From April 1991 all NHS organisations, including the PHLS from April 1992, had to pay an annual charge—the ‘capital charge’—on land, buildings and equipment that were subject to depreciation. The charge was calculated at current capital cost and not historic costs, and had to be paid from annual revenue and returned to the Treasury. It was made up of public dividend capital (similar to equity) and interest-bearing debt, equivalent to Treasury loan stock. The government expected that using private-sector accounting techniques for capital would encourage managers to make better use of physical resources and enable comparisons with the private sector.^49^

As the PHLS had neither public dividend capital nor Treasury loans, a notional cost of capital was calculated at 6 per cent of the average capital employed and charged to its income and expenditure account.^50^ While the block grant that the PHLS received from CFS for its central laboratories covered their notional capital charge in full, peripheral laboratories had to first estimate and then build their capital charge into the prices or tariffs that were being negotiated for services.^51^ This would increase their costs, which would need to be passed on to the hospitals. And while the PHLS had direct management of the laboratories, it did not own the estate of those laboratories belonging to the NHS trusts. Each NHS trust would likewise have to pass on a portion of its laboratory capital charge to the PHLS, which in turn again included it in its prices for contracts with the region.^52^ Because the sums allocated to district health authorities were based on average, not actual, costs, laboratories with higher costs such as those in London or those with recent capital investment would be disadvantaged.^53^

Then there was the issue of costing services and tests. It had already been noted in 1987 that there was insufficient management information to allow laboratories to charge health authorities. In 1991, in the first of three reports on pathology services, the Audit Commission described the difficulty laboratory managers faced in getting to grips with the complexity of pathology costs—in effect, the cost of each diagnostic test—not just in the PHLS but in the 1,200 NHS laboratory departments (biochemistry, haematology, microbiology, histopathology, etc.) within the approximately 300 NHS laboratories in England and Wales. Estimation of the full cost of these services was not straightforward, because the PHLS, NHS, and many university laboratories shared laboratory space, resources, staff and equipment.^54^ Informal agreements were widespread. Now there was a need to understand the full costs of the old cost-sharing arrangements before trusts or provider units would be able to review service requirements and understand any additional costs to ensure that they were fully funded.

The need to unbundle and separate out the staff, overheads and costs of diagnostic services as part of the internal market undid decades of carefully established working arrangements between the PHLS and the NHS. It hindered the flow of necessary public health information to the PHLS and introduced an enormous market bureaucracy. Staff were diverted to creating inventories of goods and services, assigning facilities ownership and then making detailed charges in both directions. Additionally, the PHLS had to reassign staff to negotiate contracts with all its customers.

Local arrangements and goodwill were undone, especially as managers felt the pressure on revenue budgets, and negotiations were not always cordial. An added frustration for the PHLS was that many trusts simply didn’t make the agreed payments, so further resources had to be diverted to credit control. Despite all this, in 2000 DH officials noted that the PHLS claimed it still ‘subsidises’ NHS microbiology services, by £12 m a year out of core funding of £55 m, down from around half the costs of the laboratories in 1994.^55^ In reality this ‘subsidy’ was simply the cost of running the public health service of the laboratories.

### The effect of contracting for peripheral laboratory services

The change in PHLS sources of income can be clearly seen in Figures 1 and 2. Total PHLS income (excluding capital grants) rose following the introduction of the internal market and transfer of peripheral laboratories from the NHS, from £62.8 m in 1990/91 to £152.3 m in 2002/03. Much of this increase in the first two years was due to the transfer of staff to PHLS direct management from 48 health authorities and NHS laboratories by 1992/93.^56^ More significant to note, is the change in sources of income, with CFS income falling as a proportion of total income and income generated through rechargeable services (mostly indirectly from HCHS) rapidly increasing in cash terms and as a proportion of total income in just 2 years, from £9.0 m (14 per cent) to £44.9 m (42 per cent) of income in 1991/92 and 1993/94 respectively.

When the new system of contracting came into effect, the PHLS’ 48 English laboratories had between them (in October 1992) 668 purchasers to contract with. For instance, Newcastle public health laboratory, based at Newcastle General Hospital, had 17 customers: 12 district health authorities, a mental health trust, an acute trust, a community health trust, a hospital trust and GP fundholders. Tooting public health laboratory, on the other hand, at St George’s Hospital, had nine customers, including Lambeth health authority, Merton & Sutton Trust, Royal Marsden Hospital, and GP fundholders in Wandsworth.^57^

David Flory, chief executive of Newcastle and North Tyneside Health Authority, in his 1999 financial review of the PHLS for the DH (see below), noted huge variations in contracts and widespread differences and inconsistencies in charging arrangements, sometimes simply reflecting the different negotiating strengths of the trusts and the PHLS.^58^ His recommendations focussed on improving the quality and skills of PHLS staff carrying out negotiations for perhaps 300 or more contracts with trusts and he suggested there should be a national framework with the NHS Executive for service agreements or that they should be made directly with health authorities and their primary care groups, or with NHS regional offices.

A draft memorandum to Dorrell noted, ‘the measures provided for in the NHS and Community Care Act were designed primarily to reform the provision of patient care, not the nationwide monitoring and prevention of disease.’^59^ Contracting and charging for public health services introduced new anomalies and new tensions with local authorities and NHS laboratories. Although local authorities were not obliged to use PHLS laboratories, they generally did so because they were not charged and the quality of the work was high. In 1992, about 4 per cent of the peripheral laboratories’ workload was for environmental health officers, for whom the service was fundamental. The DH considered it reasonable that the PHLS not charge for work that stemmed from disease outbreaks or that it had other specific funding to undertake. However, from 1992/93, while the PHLS continued to provide for free the services it had been doing up until then, it began to charge for any additional work, for which it would receive no DH funding.^60^

This angered local authorities, which had barely been consulted, and puzzled the PHLS’ own laboratory directors. The director of the Chelmsford public health laboratory wrote to the chief environmental health officers he worked with that

*it seems that this laboratory will be able to provide a ‘free‘ service for each EHD [environmental health department] up to the level of activity of that EHD for the FY 91/92. Above that level of activity, we will have to charge. … There is awareness at national level that there is an intrinsic unfairness in these arrangements. EHDs which have historically sent few specimens, perhaps as a result of underfunding, will have to bear an extra financial burden if they need to increase their activity levels in response to new legislation.* ^61^

Contracting also created tensions with the 300 or so NHS laboratories outside the PHLS network of about 48 jointly run PHLS and NHS laboratories. They felt it was unfair that there was no support from the DH or health authorities for the wider surveillance work. These laboratories had been pleased to send data to the CDSC, often in informal arrangements receiving benefits in kind from the PHLS, such as for supplies of PHLS reagents or membership of the Colindale-run accreditation scheme, NEQAS.^62^ They considered that they were doing the same work as the PHLS, to the same standards, and making important public health contributions. The Association of Medical Microbiologists and the Association of Clinical Microbiologists noted that to ensure that no epidemiological information was lost, the PHLS might require considerable funding, and that this would amount to unfair competition with non-PHLS laboratories.^63^

Prof. Dick Madeley at the Royal Victoria Infirmary in Newcastle complained in January 1992 that his laboratory had identified about 40 per cent of the isolates of influenza A in England and Wales, but he could not continue with this work in a market system if the costs were to be passed on to customers, as he would not get the subsidy from the DH. ‘Without urgent action an important part of national virological surveillance may crumble and, once lost, it will take literally years to rebuild.’^64^ In a coordinated campaign, many other consultant virologists wrote to the CMO with similar concerns, and met with a sympathetic response from the DH and PHLS, though there was little to be done, except to say that health authorities had a local responsibility for public health.^65^

The DH, recognising that decreasing hospital requirements for surveillance could threaten the PHLS’ surveillance data, suggested that ‘the labs should be free to adjust their contracts so as to give hospitals a financial incentive to continue referring specimens at the levels required by the PHLS’, thereby undermining an internal market principle that contracts must reflect only the charges incurred, and not any other financial incentive or discount.

The need to ensure that data continued to flow to the CDSC was also highlighted in the Abrams review of public health, which was communicated in an NHS Management Executive circular of November 1993, *Public health: responsibilities of the NHS and roles of others*. It reminded all parties in communicable disease control that surveillance was dependent on a free flow of information to consultants in communicable disease control (CCDCs) in the health authorities and the CDSC, and all parts of the NHS should accept this. ‘This acceptance should be reinforced in the contractual process which in particular should ensure that relevant laboratory test results are passed on in a timely manner to CCDCs and CDSC’.^66^ In March 1994 the CMO, Kenneth Calman, was briefed to advise the PHLS that the government intended to place a statutory duty on all laboratories to notify clinical results.^67^ Although this was repeatedly stated in subsequent years, the change was not enacted until the Health Protection (Notification) Regulations 2010.

Other pressures weighed on the PHLS. In an attempt to generate economies of scale, the network of peripheral laboratories was restructured between 1994 and 1996 into 10 regional groups. These were broadly based, the PHLS said, on existing collaborative arrangements, and were largely similar to NHS management regions introduced in 1996. Up until then, each of the 52 or 53 laboratory directors in England and Wales had reported directly to the PHLS director.^68^ This was, as Graham Hart noted, ‘not a credible management arrangement’.^69^ The PHLS expected that it would allow a redistribution of the analytical work between sites, enabling rationalisation of service, yet ‘not give rise to the same degree of opposition as total withdrawal from some laboratories’.^70^ The plan mirrored the ‘hub and spoke’ pathology model that was being introduced in England at that time as part of an overall development of laboratory restructuring known as pathology modernisation. In this model, laboratories were grouped into small networks, in which one was the hub, coordinating the others and carrying out the bulk of routine work. The spoke laboratories were closer to or on hospital sites and undertook urgent work. Specialist skills could be shared across networks. For the PHLS this in particular meant ensuring that skills and knowledge in its reference and specialist laboratories were maintained, where they were located away from Colindale in peripheral laboratories.

In 1996, the PHLS closed three laboratories: Bath, Guildford and Wolverhampton. The NHS laboratory contracts with Bath were renegotiated and Bristol laboratory took over the contracts; staff at Guildford were transferred to Frimley NHS trust; and the PHLS laboratory at Wolverhampton was transferred to the Wolverhampton hospital trust.^71^ The DH suspected that these closures were simply a way of passing costs elsewhere in the NHS.^72^ Nevertheless, and quite remarkably, in the face its financial pressures, the PHLS managed to retain 46 laboratories (Table 3).^73^

**Table 3.** PHLS, HPA, PHE and UK Health Security Agency laboratories—selected years, 1961/62–2021/22

	Reporting year ending											
	PHLS							HPA		PHE		UKHSA
	1962	1970	1974	1978	1981	1995	2003	2007	2012	2015	2020	2022
Central laboratory—reference & specialist units	10	11	14	11	9	9	7					5
Special laboratories	4	5	3	4	5	1	2					
Peripheral laboratories	59	63	59	52	52	53	46					
Regional and collaborating NHS laboratories								44	22			^a^
Public health laboratories										8	^a^	
CAMR, Porton Down—research & reference units					9							
Total	73	79	76	67	75	63	55	44	22	8	^a^	^a^

^a^Not published.

Sources for figures and tables

Public Health Laboratory Service Board, *Accounts 1974–75*, 1976; *Accounts 1975–76*, 1977; *Accounts 1976–77*, 1978; *Accounts 1977–78,* 1979; *Accounts 1978–79*, 1980; *Accounts 1979–80*, 1981; *Accounts 1980–81,* 1982; *Accounts 1981–82*, 1983; *Accounts 1982–83,* 1984; *Accounts 1983–84*, 1985*; Accounts 1984–85*, 1986; *Accounts 1985–86*, 1986; *Accounts 1986–87*, 1988; *Accounts 1987–88*, 1989; *Accounts 1988–89,* 1990; *Accounts 1989–90*, 1991; *Accounts 1990–91,* 1992; *Accounts 1991–92,* 1993; *Accounts 1992–93,* 1994; *Accounts 1993–94,* 1995; *Accounts 1994–95,* 1996; *Accounts 1995–96,* 1997; *Accounts 1996–97,* 1998; *Accounts 1997–98,* 1999; *Accounts 1998–99,* 2000; *Accounts 1999–2000,* 2001; *Accounts 2000–01,* 2001; *Accounts 2001–2002*, 2003; *Accounts 2002–2003*, 2004.

Health Protection Agency. *Annual Report and Accounts 2006*, 2006; *Annual Report and Accounts 2011/12*, 2012.

McCartney, ‘Regional Microbiology Network’.

Public Health England, *Annual Report and Accounts 2014/15,* 2015; *Annual Report and Accounts 2019/20,* 2020.

UK Health Security Agency, *Annual Report and Accounts 2021/22*’, 2023.

HM Treasury, *GDP Deflators at Market Prices, and Money GDP December 2023 (Quarterly National Accounts)* (8 January 2024). https://www.gov.uk/government/statistics/gdp-deflators-at-market-prices-and-money-gdp-december-2023-quarterly-national-accounts

## The Road to a Communicable Disease Strategy and the Demise of the PHLS

In 1999 the public health minister Tessa Jowell asked the CMO Liam Donaldson to develop a communicable disease strategy. Part of the context for the strategy was the increasingly strained relations between the PHLS and the DH. There had been concerns about PHLS management since at least the time of Donald Acheson. The disagreements about budgets and the Flory report had heightened tensions. David Flory, writing with data analyst Wendy Jones and future Welsh CMO Ruth Hussey, found inconsistencies in planning between central and local levels, ‘a lack of financial expertise and skills within the group structures, and insufficient central support’, a lack of commitment by some group directors to balancing income and expenditure, and most seriously variations in financial projections between, for instance, the Finance and General Purposes Committee in March 1999 showing a budgeted deficit of £679,000, the accountability review meeting the same month showing a £900,000 deficit, and a projection in August of over £1m deficit, with £1.3 m in contingencies.

*The existence of different projections of this kind produced so close together gives cause for serious concern and undermines confidence in the robustness of the financial forecasting within the organisation. … Does the organisation simply not know its financial position at any one time? Does it know but chooses in different documents to disguise the true position?* ^74^

The DH accepted the findings of Flory’s report, noting that while PHLS director Diana Walford and chairman Sir Leslie Turnberg did not appreciate the problems, neither did their own staff working with the PHLS, and that there were lessons for them in how to deal with arms length bodies.^75^ The PHLS, however, vigorously rejected the criticisms, claiming that its unpredictable income was due to unreasonable demands for cost savings from trusts and their failure to sign contracts in a timely manner if at all.^76^ Flory had in fact, as noted above, recognised these problems in the contracting process (see *Getting ahead of the curve* section), and recommended improvements in the contracting process, which were only partly acted on by the DH.

On 5 May 2000, Andy Smith, in the DH public health division, wrote to the deputy CMO, Pat Troop, the senior departmental sponsor of the PHLS, that the Board ‘clearly felt angered over what it saw as discourteous behaviour from the Department’, citing Jowell’s successor Yvette Cooper ‘setting aside only one hour for the Review meeting and leaving before the end’, repeated postponements of the meeting with Cooper, the handling of the Flory report, and the failure of the DH to implement Flory’s recommendations on improved contracting with trusts and health authorities.^77^ The board minutes were if anything more strongly worded:

*the way the Service was being treated was intolerable. The continued failure to meet deadlines, the repeated evasion of responsibility by the NHS Executive and the lack of support from the PHLS’s sponsor department were a cause of grave concern. … It seemed that although the government clearly needed the PHLS, it was unprepared to pay for it.* ^78^

Board member Pamela Taylor, chief executive of Water UK and a past president of the Institute of Public Relations, demanded a meeting with the NHS Executive and wanted ‘a media and political campaign’ to raise the importance of the PHLS.

From the point of view of John Bywater, head of the DH unit dealing with the PHLS and other non-departmental public bodies, writing to the permanent secretary Chris Kelly in May 2000,

*There is a general tendency for the PHLS to expect others to solve their problems which might account for why we are not totally sympathetic to what they tell us. Sir Leslie no doubt feels that we and Ministers should accept at face value whatever the PHLS Board tell us. From experience in seeing the Board in action I find it difficult to accept that any decisions made by them are a result of a real understanding of the issues.* ^79^

Turnberg had, he said, enjoyed an open-door relationship with Jowell, and thought officials prejudiced Cooper against the PHLS. He’d only agreed to remain as chair, Bywater continued, if the DH stopped being so ‘hands on’. But the PHLS was putting forward plans ‘which failed to set out the financial position as well as how anticipated deficits will be handled’ and he had previously written to Troop, ‘We must ensure that they do not go ahead with their plans before we have approved the strategy and any business cases arising from it’.^80^

The strategy for communicable diseases *Getting ahead of the curve,* published in January 2002, described the ongoing challenge of communicable disease, in particular tuberculosis, health care-acquired infections, antimicrobial resistance and blood-borne and sexually transmitted infections, as well as the new threats of emerging diseases and bioterrorism.^81^

Among the actions proposed by the report to implement the communicable disease strategy were two involving structural change to health protection services. Firstly, a single agency would be created to address concerns that health protection was fragmented by providing national expertise and key services at national, regional and local level. Its functions would be not just to protect against infectious disease and to prevent its spread, but also to protect against other dangers to health, including chemical, radiological and bioterrorist threats. It would provide ‘a clear line of sight from national to regional to local level for the health protection function’.^82^ The new agency—the Health Protection Agency (HPA)—would go on to absorb 80 organisations, among them the PHLS, which would be abolished.

Secondly, with the PHLS abolished, the public health and hospital microbiology laboratory network would be restructured. The report considered that the PHLS was ‘organisationally complex’, consisting as it did of three strands: (i) eight groups of laboratories, (ii) the reference laboratories and (iii) the CDSC. The microbiology laboratories formed a ‘fragmented system’ with a variety of management arrangements. A single point of management would achieve better co-ordination and enable improvements in standardisation of procedures and the introduction of new technology. Laboratories should be clearly categorised into those providing routine diagnostic microbiology work and those providing public health, specialist or reference functions.^83^

While the PHLS had been actively involved in the communicable disease strategy, the decision to abolish it and transfer its laboratories to NHS trusts came as a surprise to the PHLS board. Turnberg had reported to the board in May 2000 that in a previous conversation, ‘the CMO had emphasised that the communicable disease strategy would not be proposing the break-up of the PHLS’.^84^ For a few years the DH had seen papers about changing the status of the PHLS, including in early 2000 separating out the CDSC, of which the PHLS was probably aware.^85^ But Turnberg and Walford were not informed by the CMO until less than a week before the report’s publication, and now described the decision-making as ‘secretive, opaque and lacking any visible process for systematic, informed input’.^86^

This time PHLS was on the back foot and without the direct route into the minister. Following the announcement of the recommendations for the abolition of the PHLS, the director of the US National Center for Infectious Diseases in Atlanta (part of the US Centers for Disease Control and Prevention), Dr James Hughes, wrote to PHLS board chairman Turnberg as follows:

*reducing the number and capacity of existing public health laboratories under the proposed reorganisation may be counterproductive. The flow of information between the public health and the clinical sectors will likely be reduced, as will the ability of public health agencies to leverage new information into changes in laboratory practice and policy on a broad scale … I suggest that you carefully assess the potential ramifications of the changes in laboratory services.* ^87^

Turnberg may have been instrumental in creating a sub-committee of the House of Lords select committee on science and technology, of which he was a member, to carry out an inquiry into infectious disease. It was established in May 2002 in response to *Getting ahead of the curve*, and also perhaps because of the break-up of the PHLS. It made a call for evidence on, among other things, disease surveillance and the report’s recommendations. Prof. Hugh Pennington was one of a number of respondents critical of the decision regarding the PHLS and supporting the CDC’s comments:

*The transfer of PHLS laboratories to the NHS is being done at breakneck speed. The reasons for this, or its urgency, are unclear. The impact on surveillance will be negative. The transfer will destroy a network of laboratories with the public health function at their core. It is improbable in the extreme that NHS hospital managers will give this function the priority that it received from the PHLS. Inevitably, they will focus on diagnostic work for patient management rather than surveillance or outbreak control. The training function of the PHLS will disappear. This will be a major loss to UK medical microbiology: doctors and scientists with expertise, experience, and an interest in public health microbiology will in future emerge only capriciously and randomly.* ^88^

Others agreed that the speed and the lack of formal review was baffling. They found it hard to see the merits of the HPA commissioning but not managing all required laboratories, and noted that arrangements were constrained by the previous ‘serial disorganisations’ of health services. The break-up of the network would interrupt the complex interaction between specialist, reference and peripheral laboratories. The strengths of the PHLS system included the flow back and forth of public health and clinical information through the combined NHS/PHLS laboratories and a combination of epidemiology and public health microbiology. There was a synergy between FW&E microbiology for local authorities and clinical microbiology for hospitals and GPs. There were now ‘legitimate concerns about whether the capacity within both the HPA and local departments will be sufficient to respond to surges’ ie, outbreaks and epidemics, and to carry out enhanced surveillance. As for the transfer of the laboratories to the NHS, according to Sheffield City Council, NHS-managed laboratories had ‘a poor understanding of their wider public health responsibilities’. If the public health function of NHS laboratories was not clearly identified and funded and made a statutory responsibility, NHS hospital managers would focus on diagnostic work for patient management rather than surveillance or outbreak control.^89^

## A policy without a clear rationale?

The haste and speed and lack of any formal review and consultation prior to the abolition of PHLS raises the question as to what the policy rationale was for the abolition of the PHLS. The main reason given at the time was complexity and fragmentation. In other words, its authors thought it to be a rationalisation. A 2013 HPA witness seminar involving key decision makers at the time provides some insights into the new mindset of the civil service and the medical workforce within it. It also provides insight into how market ideology resulted in an increasing need to check the independence of agencies.

First, the decision not to consult PHLS on its abolition until after the review and recommendations had been made was greatly at odds with the careful and detailed consultations that were the hallmark of earlier reviews. Donaldson said at the seminar that he and Troop produced the strategy having taken away ‘the wisdom, ideas, views and proposals’ from a committee he had established.^90^ This may explain the criticisms of secrecy and undue haste.^91^

The political ideology of the Conservative governments of Margaret Thatcher from 1979 and John Major from 1990 was now inculcated within the civil service. Market-oriented reforms and increased managerialism—‘new public management’—focussed civil service minds on economy, efficiency, and effectiveness (as opposed to policy advice and co-ordination), and on the greater use of performance measurement. These developments were continued under the Tony Blair administration, with public service agreements creating efficiency targets for departments and investment only ‘with strings attached’, the strings of reform and modernisation.^92^ ‘More business-like’ management practices from the private sector were introduced. Agencies and non-governmental organisations, created to deliver policy, were now seen as unaccountable and lacking ‘political antennae’.^93^ The PHLS, though created in legislation as an independent body, was funded by the DH, and its activities and expenditure were ultimately part of the DH’s public service agreements. Civil servants having to deliver these agreements may well have been frustrated by any resistance from the PHLS, especially if they had come to understand public service through the lens and ideology of new public management and marketisation.

The fact that the PHLS was simply exercising its statutory independence, to which its board members resolutely clung, brought it into conflict with the DH, sometimes publicly, as with the DHSS’ proposed reforms in the 1980s. Independence might now be viewed as unwelcome opposition, leading to a wariness about consulting with it. The PHLS was not the only organisation asserting its independence. A similar fate befell the National Radiological Protection Board (NRPB), which like the PHLS was taken unawares by the announcement of its abolition.^94^ William Stewart, the first chair of the HPA, recalled in the 2013 seminar that when the NRPB chairman wrote to ministers requesting continued independence, ‘the result was I became chair of the NRPB and Shadow Chair of the HPA’.^95^

A second reason given by Donaldson was that ‘it was felt by the finance people in the DH that the PHLS had gained increases in funding relatively easily’, and he considered that in the HPA ‘there would be a much more business-like approach towards determining fair resource allocation’. Pat Troop, Donaldson’s deputy in 2003 and the first chief executive of the HPA, agreed that ‘the traditional public health side’ and the PHLS ‘felt that if they needed the money for what they were doing, the Government should provide it. It was a moral issue. They did not feel they should go out to earn extra’. As HPA finance director, Tony Sannia, commented, ‘it is probably a false division between external income and GIA [grant in aid]. All money is equal and it all comes from somebody who wants something in return for that money’.^96^

Donaldson’s account of PHLS funding is inconsistent with the well-evidenced pressures on CFS funding, with government grants accounting for just 40 per cent of its revenue income in 2002/03.^97^ Moreover, Donaldson’s 2005 and 2006 annual reports stated that public health budgets were ‘raided’ to reduce hospital deficits or to meet the demands of acute healthcare, that is, of patients.^98^ Why the deputy CMO thought the PHLS should ‘earn’ funding for public health surveillance and other health protection activities was never explained. But this gives some insight into the new mindset of career civil servants, including its medical staff.

There was a perception that PHLS was ‘looking back to the ‘golden age’, of the Public Health Laboratory Service’, according to Stephen Gillespie, HPA’s London regional microbiologist.^99^ This did not fit with the government ideology that was pushing hard for competition and greater private-sector involvement in the NHS, including in laboratory services. The PHLS already had to compete with private laboratories to provide microbiology services to trusts. In response to a suggestion in a 2002 Health Select Committee report on the role of the private sector in the NHS that ‘a variety of models needs to be tested’ to upgrade laboratories and improve services, the government emphasised only the importance of using the independent sector, which had ‘expertise and resources … to develop cutting-edge new technologies and tests … [and] expertise in process re-design, procurement procedures and project management …’.^100^ Just as market-derived approaches drove civil service behaviours from Thatcher onwards, so they drove health policy on into the Blair premiership which kept in place the internal market and associated changes, and saw the private sector brought in to play a bigger role under-secretary of state for health Alan Milburn.^101^ The disbanding of the PHLS was proposed not in a white paper but in a report by Donaldson and Troop. Donaldson himself was very much in agreement with Labour’s policies, and personally advocated franchising NHS hospitals to not-for-profits and management and other private sector companies.^102^

A third reason was professional rivalry within the PHLS and between it and the NHS. According to Donaldson, epidemiologists in the PHLS felt ‘undervalued compared to the laboratory side’. He himself wanted epidemiology to take a more important role in the PHLS, and felt that the abolition was an ‘opportunity to rebalance things’, although as Mike O’Brien, a member of the 1988 Acheson review of the future of public health and a regional medical director, had pointed out in 2002, ‘the solution lies in management action, not in the creation of a new Agency’.^103^ That the PHLS and those with whom it interacted suffered in particular from medical and professional demarcations that hampered cooperation internally and externally had been observed for some time. Turnberg was advised in 2000 by Troop’s predecessor, Eileen Rubery, to, in his words, ‘strengthen relationships with others and engender a positive image’.^104^ The Flory report commented on it and noted that there should be more public health input into senior management.^105^ As far back as 1994, the senior medical officer Elizabeth Tebbs had written, ‘I see almost daily evidence of “us and them” attitudes of microbiologists and epidemiologists … A lot more could be done to improve collaboration between the two groups throughout the PHLS.’^106^ In 1999, the PHLS had to withdraw from its laboratory in Oxford, where it had established its first regional epidemiology post, because of professional disagreements. Local clinicians ‘did not share the PHLS view of the public health philosophy and were anxious that pathology should be headed by an infectious diseases physician rather than a microbiologist.’^107^ The PHLS management accepted there was an issue, and up to 2002 had been developing combined units where epidemiologists and microbiologists could work together on specific areas, such as gastrointestinal infections.

For some, though, the new restructuring created the danger of a schism between microbiologists and epidemiologists. According to David Dance of Plymouth Hospitals, ‘the integration of microbiology and epidemiology that encapsulates the PHLS model has long been regarded as one of the strengths of the system’. Rather than redressing an imbalance between the two professions, it seemed to Sheffield City Council that the HPA proposals followed ‘a strategy of divesting all clinical microbiology … out of its management, as if clinical microbiology has no core Public Health function’.^108^

Tebbs had also noticed in 1994 that the PHLS’ claim to be ‘uniquely placed’ to carry out surveillance of hospital-acquired infection ‘overstates the case and will annoy many NHS microbiologists’. The following month Peter Kendall at the DH reminded Walford that there is ‘a narrow but important line between recognising and reinforcing PHLS’ uniqueness and expertise … and implying that its uniqueness and excellence is such that PHLS has a monopoly on the provision of services in the control of communicable disease and infection … The Department would find it helpful to have the relationship between PHLS and other public health providers examined in more detail’.^109^ The idea that it was an ‘Old Boys’ network lingered.^110^ The ongoing issue of tensions between the PHLS laboratories in the NHS was ‘a really big and controversial issue’, according to Donaldson. Gillespie described ‘a considerable degree of hostility from the NHS towards the PHLS and the microbiology service’, which both he and Donaldson claimed the merger addressed.^111^ But professional rivalries are common within and across health services and are not sufficient reason for a radical restructuring.

None of these reasons apart from the imperatives of market ideology are sufficient to explain the decision to abolish the PHLS. More likely the complexity of market contracting for a public health service had proved too problematic for civil servants and highlighted all the negative public health effects of contracting, the fragmentation and complexities of dealing with multiple purchasers and providers, and the purchaser/provider split that public health now found itself straddling.^112^ And so a decision was made to create a centralised agency ostensibly along the lines of the Centers for Disease Control and Prevention, as in 1985. Indeed, the report’s title echoed the 1995 article *Emerging infections: getting ahead of the curve* by its director David Satcher.^113^

### Epilogue

The HPA was set up in England and Wales on 1 April 2003, and was established by statute in 2004 as a UK-wide non-departmental public body from 2005. For the first time, controlling the spread of infectious disease became the statutory function of a centralised public body which, combining the different aspects of health protection, considered itself to be ‘a global first’.^114^

At the time of the creation of the HPA, the PHLS’ public health laboratory network consisted of 46 laboratories, the CPHL with seven reference laboratories and seven collaborating laboratories in London, including at the London School of Hygiene & Tropical Medicine and University College Hospital. In 2003, the PHLS, winding itself down ahead of its legal abolition on 1 April 2005,^115^ transferred the central and reference laboratories and the CDSC with its surveillance functions into the HPA, along with each of the regional laboratories.^116^ The management of 32 of the remaining laboratories was transferred to the NHS trusts where they had been based,^117^ alongside the 330 existing NHS clinical microbiology laboratories in England. Their funding and commissioning were transferred to the 300 or so recently created NHS primary care trusts (PCTs), which replaced health authorities and which could exercise the secretary of state’s power to provide a microbiological service. The HPA, meanwhile, managed the CPHL and most of what had been the PHLS’ specialist and reference laboratories so as to provide co-ordination and specialist testing.^118^

Central to *Getting ahead of the curve* was the ‘rationalisation’ of microbiology laboratories. What had been seen as a strength—the blending of hospital and public health microbiology—was now regarded as an incongruity. The organisational arrangements required to deal with a wide range of surveillance functions were understood as unnecessary complexity. However, with the split between purchaser and provider still firmly in place, the HPA now had to enter into contracts or service-level agreements with 37 collaborating laboratories, which included all those transferred from the PHLS to the NHS. These were to commission services for public health testing, general public health surveillance and support activities and local authority FW&E analysis. The HPA had to make separate funding and service agreements with each of the 300 PCTs for clinical microbiology.^119^

These collaborating laboratories were also, like most of the other clinical diagnostic microbiology laboratories, managed by NHS trusts and foundation trusts, commissioned by PCTs for clinical diagnostic microbiology. Ringfenced funding was given to PCTs for the laboratories but only until April 2005, after which any extra funding to cover their new responsibilities for public health microbiology would be diverted from national public health to local trusts, where it would compete with other priorities.^120^

In 2004, the post of Inspector of Microbiology and Infection Control was created, and Prof. Brian Duerden, deputy director and briefly director of the PHLS, appointed to it. He was to be the only appointee. The role was to ensure that NHS diagnostic laboratories continued to contribute to public health microbiology and to develop consistent standards across laboratories. Duerden worked with the HPA to create the Regional Microbiology Network comprising eight regional microbiology laboratories and 36 collaborating laboratories, along with 26 FW&E laboratories, nine of which were HPA-managed and 17 in NHS trusts (Table 3).^121^ However, the commissioning arrangements cut across the network.

The number of HPA and NHS collaborating laboratories decreased from 44 in 2007 to 22 in 2012. In 2008/09, the HPA’s government funding was cut in real terms following the global financial crisis. The Carter review in 2006 noted that PCTs had no obvious accountability for public health, and the new foundation trusts were too competitive to work cooperatively.^122^ Under the HPA’s successor from 2013, Public Health England (PHE), an executive agency of the DH responsible for implementing the government’s legal duty imposed in the Health and Social Care Act 2012 to protect against health threats, the network seems to have broken up entirely, with only eight laboratories under its management (see Table 3).^123^

In a letter to The Times in August 2020, Turnberg lamented, ‘Our disastrous inability to deal with the Covid-19 crisis can be traced back to 2004 when the government decimated the nationwide network of public health laboratories overseen by the Public Health Laboratory Service … Little wonder we are ill equipped to deal with the virus.’^124^

In June 2020, Duerden and other senior medical microbiologists, reflecting on the state of public health microbiology, had noted that, as predicted, NHS trusts had indeed focussed on local clinical needs rather than public health surveillance, and their laboratories and cooperation had been diminished.^125^ They went on to state that training for microbiologists lacked sufficient epidemiology or control of communicable disease. Furthermore, professional rivalries had not been resolved, and tensions between the laboratories and surveillance epidemiology had continued.^126^

According to Duerden and colleagues, plans to create regional laboratory networks as part of the pathology modernisation programme were still incomplete in 2020 and the service remained fragmented and lacking in coherence. The reorganisation had specifically aimed to address fragmentation, although at the time critics warned that the laboratory service would no longer function as a whole. In particular, national outbreak capacity was damaged and surge capacity had been lost, as anticipated by the Faculty of Public Health Medicine.^127^ By 2020, it was not even clear who was responsible for large-scale testing–but not PHE, according to its chief executive.^128^

England found itself, when Covid-19 arrived, without an established network, strong links between universities and public health laboratories, or even a clear understanding of laboratory and staff capacity,^129^ It was forced, therefore, to set up a totally new system of laboratories, unaccredited and of necessity in partnership with universities and the private sector, known as Lighthouse laboratories, with all the problems that followed.^130^

## Conclusion

The PHLS was established at the same time as the NHS in post-war England when infectious disease was an everyday threat to life. It was led by independent men with strong personalities who were part of a scientific and medical hierarchy and who were regarded by some as forming an old boys’ club. It worked alongside a civil service, the members of which were focussed on policy advice and co-ordination.

Both the PHLS and the civil service were to be significantly impacted by major shifts in economic policy from the late 1970s onwards, alongside an increased public health focus on individual behaviours.

The PHLS suffered a series of expenditure cuts, beginning first under the Labour Callaghan government following the International Monetary Fund crisis in 1976, and continuing under Margaret Thatcher’s Conservative government first elected in 1979 and its neoliberal economic policy. The policy required civil servants to become more managerial, focussed on efficiency and value for money, and to internalise business and market values.

Meanwhile, PHLS grant funding, unlike NHS funding, was not uplifted annually in line with inflation, and contrary to Treasury guidance it was not passing on to NHS hospitals and local authorities its costs of providing them with laboratory services.

These pressures caused tension in the relationship between civil servants and PHLS staff, and led to attempts in the 1980s to break up the network by transferring all of its peripheral laboratories to the NHS. These were successfully opposed with the argument that public health needs would be trumped by an inevitable NHS focus on clinical needs.

However, once the internal market was introduced in the early 1990s, and its grant funding reduced, the dissolution of the PHLS was again on the cards. Recognition that the interests of public health were not the same as the aggregated interests of individual customers was not to be a winning argument. A new financing landscape dawned and the strengths of the PHLS, such as scientific and medical cooperation between fellow public bodies and independent advice, now had to operate within market strictures, such as the capital charging regime and the purchaser/provider split. This ultimately proved to be unsustainable, and the PHLS model was dismantled in 2003 with its peripheral laboratories transferred to the NHS and its central laboratories taken over by a new HPA following a rapid and ill-thought through reorganisation. The HPA was in turn abolished in 2013, replaced by PHE, which was also then replaced in 2021 during the Covid-19 pandemic by the United Kingdom Health Security Agency.

Nearly 20 years passed between the dismantling of PHLS, and the start of the pandemic. During that period, the NHS, as well as the public health system, were subjected to several further reforms, and were significantly impacted by austerity policies following the 2008 financial crash. Lack of public health laboratory capacity was not identified as a deficiency in the response to infectious disease outbreaks that occurred before the pandemic, such as SARS in 2003 and swine flu in 2009, presumably because they were, fortunately, comparatively modest in scale. And following the reported emergence of a novel coronavirus in China in late December 2019, PHE was quick off the mark the following month in developing a test.^131^ Yet an established and extensive public health laboratory network is an essential component of a well-functioning, multi-faceted public health system that is capable of providing an effective outbreak response, and as Kirchhelle has pointed out, on the eve of the pandemic ‘the once formidable public health laboratory infrastructure in England was smaller than at any point since 1939’. In the light of Covid-19, those who would argue that it was wise to break up that infrastructure have a hard case to make.

## Footnotes

1 Department of Health and Social Security, *The Future Structure of the National Health Service* (London: Department of Health and Social Security, 1970), 1–2.

2 Peder Clark, ‘“Problems of Today and Tomorrow”: Prevention and the National Health Service in the 1970s’, *Social History of Medicine*, 2020, 33, 981–1000.

3 For instance, Virginia Berridge, D. A. Christie and E. M. Tansey, eds, *Public Health in the 1980s and 1990s: Decline and Rise?* Vol. 26 (Wellcome Witnesses to Twentieth-Century Medicine. London: Wellcome Trust Centre for the History of Medicine, 2006); Alex Mold *et al*., *Placing the Public in Public Health in Post-War Britain, 1948–2012* (London: Palgrave Macmillan, 2019).

4 Infected Blood Inquiry, *The Report. Vol 5: What Happened and Why* (London: House of Commons, 2024), 27696. https://www.infectedbloodinquiry.org.uk/reports/inquiry-report

5 Claas Kirchhelle, ‘Giants on Clay Feet—COVID-19, Infection Control and Public Health Laboratory Networks in England, the USA and (West-)Germany (1945–2020)’, *Social History of Medicine*, 2022, 35, 19; Claas Kirchhelle, ‘Emergencies & Omissions – the Evolution of UK Communicable Disease Administration and Pandemic Preparedness (1939-2019). Expert Report for the UK COVID-19 Inquiry’ (2023) https://covid19.public-inquiry.uk/documents/inq000205178-expert-report-of-dr-claas-kirchhelle-dated-june-2023/; UK Covid-19 Inquiry, ‘Transcript of Module 1 Public Hearing, Dated 10 July 2023’ (2023) https://covid19.public-inquiry.uk/documents/transcript-of-module-1-public-hearing-on-10-july-2023/.

6 R. E. O. Williams, *Microbiology for the Public Health: The Evolution of the Public Health Laboratory Service 1939-1980* (London: Public Health Laboratory Service, 1985); N. Spence Galbraith, ‘CDSC: From Cox to Acheson’, *Journal of Public Health*, 1989, 11, 187–99; C. L. R. Bartlett, ‘The Communicable Disease Surveillance Centre 1977-2002: An Overview’, *Communicable Disease and Public Health*, 2003, 6, 87–96.

7 Anne Hardy, *Salmonella Infections, Networks of Knowledge, and Public Health in Britain, 1880-1975* (Oxford: Oxford University Press, 2015); John T. MacFarlane and Michael Worboys, ‘Showers, Sweating and Suing: Legionnaires’ Disease and “New” Infections in Britain, 1977–90’, *Medical History*, 2012, 56, 72–93.

8 Marion Fourcade‐Gourinchas and Sarah L. Babb, ‘The Rebirth of the Liberal Creed: Paths to Neoliberalism in Four Countries’, *American Journal of Sociology*, 2002, 108, 533–79; Martin Gorsky, ‘The British National Health Service 1948–2008: A Review of the Historiography’, *Social History of Medicine*, 2008, 21, 437–460.

9 Rodney Lowe and Hugh Pemberton, *The Official History of the British Civil Service: Reforming the Civil Service, Volume II: The Thatcher and Major Revolutions, 1982–97* (London: Routledge, 2020), 210.

10 Declan Gaffney *et al*., ‘NHS Capital Expenditure and the Private Finance Initiative – Expansion or Contraction?’ *BMJ,* 1999, 319, 48–51.

11 Lorenzo Castellani, *The Rise of Managerial Bureaucracy: Reforming the British Civil Service* (London: Palgrave Macmillan, 2018), 114.

12 Kirchhelle, ‘Giants on Clay Feet’.

13
*Ibid*., 45; Kirchhelle, ‘Emergencies and Omissions’, 83–4.

14 The National Archives DN 1/38 PHLSB papers 1982: PHLSB 82/19 Minutes of the Board: 28 January 1982, 6. All archival references are to the National Archives unless otherwise stated; Williams, *Microbiology for the Public Health*.

15 DN 1/34 PHLSB papers 1980: PHLSB 80/3 Policy for sharing of costs in joint Public Health Laboratory Service/Area Health Authority laboratories. 16 January 1980.

16 JA 397/58 Public Health Laboratory Service Board—peripheral labs: 23 January 1984. Memo. Douglas R Harris FA2C to Lynne Fosh and Mr Collingwood. Financial arrangements for transferring PHLSB service functions to the health authorities, paras 3-4.

17 Christopher Byrne, Nick Randall and Kevin Theakston. ‘The Collapse of Keynesian Welfarism 1970–1979: Heath, Wilson, Callaghan’, in Christopher Byrne, Nick Randall and Kevin Theakston, eds, *Disjunctive Prime Ministerial Leadership in British Politics: From Baldwin to Brexit* (Cham: Springer International Publishing, 2020), 51–83.

18 DN 1/38: PHLSB 82/78 Minutes of the Board: 28 October 1982, 89.

19 JA 397/131 PHLS board meetings: PHLSB 94/12. 13 January 1994. Minutes of the Board, p. 5; JA 397/135 PHLS strategic review of the PHLS 1994 (including CMO evidence): 16 November 1994. Memo. From GA Hart permanent secretary. To secretary of state. Strategic review of PHLS; JA 397/41 PHLS: co-ordination of departmental customer interests: 28 January 1998. Memo. From John Bywater PH2.4. To PHLS customer liaison team. Meeting with PHLS customers on 30 January.

20 JA 397/58: 23 January 1984. Memo. Douglas R. Harris FA2C to Lynne Fosh and Mr Collingwood, para 2.

21 JA 428/729 Public Health Laboratory Service Board minutes 1990: PHLSB 90/39 PHLS funding in the 1990s, p2.

22 JA 397/85 Effect of the NHS review on PHLS public health surveillance work – options + proposals: 12 April 1990. Memo. Miss RDB Pease to Mrs Baxter, NHS Bill: management of PHLS peripheral laboratories, para 2.

23 JA 397/85: 12 April 1990. Memo. Miss RDB Pease to Mrs Baxter, paras 2–3; A 397/58: Finance 86/6. Appendix B. Amended draft 16 January 1986. Public Health Laboratory Service Board—Funding of the peripheral microbiology laboratories, p. 6.

24 JA 397/58: Finance 86/6. Appendix B. Amended draft 16 January 1986. Public Health Laboratory Service Board—Funding of the peripheral microbiology laboratories, p. 1; 18 November 1985. Memo. R. F. Tooher to Mr Harris. PHLSB peripheral labs: funding for NHS work; JA 397/14 PHLSB papers: PHLSB 86/35 Minutes of the Board: 24 April 1986, 27(b); JA 397/85: 12 April 1990. Memo. Miss RDB to Mrs Baxter, NHS Bill: management of PHLS peripheral laboratories, para 4.

25 JA 397/25 PHLS: review 1982 of staffing, functions, and efficiency: May 1985. Report of the D.H.S.S. review of the Public Health Laboratory Service, section 4, The peripheral laboratories of the PHLS (the regional and area laboratories).

26 Kirchhelle, ‘Giants on Clay Feet’, 19.

27 JA 397/20 PHLS: review 1982 of staffing, functions, and efficiency: May 1985. Review of the PHLS consultation.

28 JA 397/27 PHLS: review 1982 of staffing, functions, and efficiency: 04 September 1985. Letter from M. G. Rinsler, Registrar, Royal College of Pathologists, to Dr E. D. Acheson, CMO, p. 1.

29 JA 397/19 PHLS: Review 1982 of staffing, functions, and efficiency: 05 October 1984. Report of the DHSS Review of the PHLS. Comments of professional advisers: Dr R. Blowers and Prof. I. Phillips, pp. 1, 4–5, 7–8.

30 V. C. Luniewska, 1991. ‘Charles Edward Gordon Smith’. Royal College of Physicians (1991) https://history.rcplondon.ac.uk/inspiring-physicians/charles-edward-gordon-smith

31 JA 428/724 PHLSB papers 1985: PHLSB 85/47 Report of the DHSS review of the PHLS, appendix 2.

32 DN 1/38: PHLSB 82/62 Review of the functions and organization of activities of the Public Health Laboratory Service. 28 October 1982, p. 2.

33 JA 428/724: PHLSB 85/81 Letter from Norman Fowler to Dr C. E. Gordon Smith, 17 October 1985.

34 JA 397/58: 04 November 1985. Memo. CW France to Dr E. Harris. PHLSB finances.

35 JA 397/58: 13 November 1985. Memo. John H James to Mrs Banks. PHLSB: funding for NHS work.

36 JA 397/34 PHLS: Accountability reviews: PHLS accountability review. 27 May 1986. Chairman’s brief, pp. 5–6.

37 JA 397/34: 01 July 1986. Letter. John Cashman, DHSS, to Dr CE Gordon Smith. Accountability review: finance and resources: 27th May 1986.

38 JA 428/726 Public Health Laboratory Service Board papers: 15 May 87. Letter. Malcolm A. Harris to Keith Saunders.

39 JA397/58: 01 March 1988. Memo. M. A. Harris HS1 to Mr Podger. Public Health Laboratory Service (PHLS) Funding—Financial responsibility for microbiological laboratory service: enclosure, para 9.

40 JA 397/85: 09 April 1990. Memo. RDB to Mr T. S. Heppell (deputy secretary DHSS), NHS Bill: management of PHLS peripheral laboratories, paras 4–5.

41 JA 397/58: 12 May 1986. Memo. MA Harris HS1 to Mr Podger. Public Health Laboratory Service (PHLS) Funding—Financial responsibility for microbiological laboratory service: enclosure, para 8; JA 397/15 PHLS—board meetings papers + minutes: PHLSB 87/52 Public Health Laboratory Service Board corporate plan 1987, p25.

42 JA 397/38/1 Public Health Laboratory Service Board—new arrangements for diagnostic work for NHS: 09 September 1987. Letter. MH Lowe, under-secretary BMA, to Dr EL Harris Deputy CMO. Public Health Laboratory Service; JA 428/729: PHLSB 90/39 PHLS funding in the 1990s, p2.

43 JA 397/84 Effect of the NHS Review on PHLS public health surveillance work—options + proposals: 20 February 1990. Memo from Keith Saunders to J Charles Dobson. Future arrangements for the funding of the Public Health Laboratory Service, sections 2, 3.

44 JA 397/85: memo, J. Gordon Brown to J. Charles Dobson, 21 August 1990: PHLS—future funding and management.

45 Public Health Laboratory Service Board, *Accounts 1992-93* (London: House of Commons, 1994), 1.

46 JA 397/85: memo, 22 March 1990 R. Dora Pease to Mr Heppell and Mrs Shirley-Quirk, OPS/SofS; memo, 21 August 1990, J. Gordon Brown to J. Charles Dobson PHLS—future funding and management.

47 JA 397/81/1 PHLS board meetings papers and minutes: PHLSB 91/15 Audit Commission. The pathology services: a management review. Attachment to item 5: PHLSB single management of PHLS area & regional laboratories February 1991. EL(91)25. Diana Walford, medical director NHSME, to regional managers of RHAs, DHAs, SpHAs, FHSAs.

48 JA 397/98 PHLS—contracts with health authorities: January 1993. Annex to FDL(93). Contracts with Public Health Laboratories, paras 5-7.

49 Jean Shaoul, ‘Charging for Capital in the NHS Trusts: To Improve Efficiency?’ *Management Accounting Research*, 1998, 9, 95–112.

50 Public Health Laboratory Service Board, *Accounts 1994-95* (London: House of Commons, 1996), 12.

51 JA 397/83 Effect of the NHS Review on PHLS public health surveillance work—options & proposals: 18 October 1989. Memo. Mr R. J. Peters FMD4 to Ms S. V. Masters NHSME. Capital charges: NHS fringe bodies.

52 JA 397/84: 02 August 1990. Memo. Mr R. J. Peters to Mr V. Green etc. Capital charges.

53 JA 428/730 Public Health Laboratory Service Board minutes 1991: PHLSB 91/32 Finance and General Purposes Committee 12 September 1991, p12.

54 Audit Commission, *The Pathology Services: A Management Review* (London: Audit Commission, 1991).

55 JA 397/211 Pathology services review: 15 February 2000 Email, John Bywater to Bryony Enser: PHLS Contracts.

56 JA 397/85: 09 April 1990. Memo. RDP Pease to Mr Heppell. NHS Bill: management of PHLS peripheral laboratories, para 3.

57 JA 397/98: 27 October 1992. Memo. J. G. Brown HEF(A) 1B to Mr Harris FCIA3 & Mr Dobson FCIA2. PHLS contracts with the NHS.

58 JA 397/266 Implementation of the Flory report: [21] September 1999, pp. 41–42; JA 397/211: email, 15 February 2000, John Bywater to Bryony Enser: PHLS Contracts.

59 JA 397/85: 21 August 1990. Memo. J. Brown to J. C. Dobson, PHLS—future funding and management, paras 9, 18.

60 JA 397/90 PHLS—work for local authorities: 02 July 1992. J. G. Brown HEF(A)1B to Mr Kendall HEF(A)1 & Mr Armstrong PS/PS(H). Public Health Laboratory Service—contractual relationship with environmental health departments; 25 September 1992. Letter. JG Brown to Association of District Councils, Association of Metropolitan Authorities, Association of London Authorities, The London Boroughs Association, The Institution of Environmental Health Officers. Proposals for the future relationship between environmental health departments and the Public Health Laboratory Service; 11 November 1992. Letter. Dr R. E. Tettmar, director PHLS, to all chief environmental health officers. Financial arrangements for PHLS laboratory support for environmental health departments.

61 JA 397/90: 11 November 1992. Dr R. E. Tettmar to all chief environmental health officers. Financial arrangements for PHLS laboratory support for environmental health departments.

62 JA 397/86 Effect of the NHS Review on PHLS public health surveillance work - options and proposals: 23 October 1992. Memo. Ms Eileen Clifford SSO HEF(M)1 to Ms A McGinty HEF(M), Mrs G. Skinner CA-OPU2, Dr E. Tebbs HP(M)1, Mr G. Brown HEF(M)1. Meeting with Royal College of Pathologists on 20 October 1992 at Richmond House.

63 JA 397/85: 28 December 1990. Prof. I. Phillips, Association of Medical Microbiologists; 25 January 1991. Julian Hodgson, honorary secretary the Association of Clinical Microbiologists.

64 JA 397/86: 29 January 1992. Letter. Prof. CR Madeley, Dept Virology RVI to Mr J Gordon Brown. Allocation of pathology costs and pricing of diagnostic pathology tests: FDL (91) 127; JA 397/87 Effect of the NHS review on PHLS’s public health surveillance work: 26 February 1992. Letter. Prof. C R Madeley to Dr David Salisbury, Safeguarding virological surveillance.

65 JA 397/86: 20 February 1992. Memo. Kathie Binysh HP(M) to Dr Elizabeth Tebbs HP(M). Allocation of costs of pathology tests performed for public health purposes.

66 NHS Management Executive, *HSG(93)56. Public Health: Responsibilities of the NHS and the Roles of Others* (1993), annex B, para 30.

67 JA 397/134 Strategic review of the PHLS 1994 (including CMO evidence): 15 March 1994. Memo. Peter Kendall to Eileen Rubery, Annex A, B3. PHLS Strategic Review Team: 24 March 1994.

68 JA 397/135: 11 July 1994. Memo. From J. G. Brown HEF(A)4. To Dr Metters DCMO, Mr Bridges HEF(A). PHLS strategic review: progress report; JA 397/132/1 PHLS board meetings—papers + minutes: PHLSB 96/17, 25 April 1996. PHLS business plan 1996/97, p11; Public Health Laboratory Service Board, *Accounts 1996-97* (London: House of Commons, 9 March 1998).

69 JA 397/135: 16 November 1994. Memo. From GA Hart permanent secretary. To secretary of state. Strategic review of PHLS, para 3.

70 JA 397/131: PHLSB 94/43 19 July 1994. Minutes of the Board, p4.

71 JA 397/136 PHLS. Strategic review of the PHLS 1994 (including CMO evidence): 22 December 1994. Letter. From Diana Walford, director of PHLS, to Mr Peter Kendall DH. Proposed withdrawals from PHLS laboratories; JA 397/132/1: PHLSB 96/09 18 January 1996. Minutes of the Board; JA 397/132/2 PHLS board meetings—papers + minutes: PHLSB 96/02 19 October 1995. Minutes of the Board, p2.

72 JA 397/136: 02 December 1994. Memo. From J. G. Brown HEF(A)4, to Mrs Marsden FCIA-FLIP2. PHLS strategic review.

73 Department of Health, *Getting Ahead of the Curve: A Strategy for Combating Infectious Diseases (Including Other Aspects of Health Protection)*. *A Report by the Chief Medical Officer* (London: Department of Health, 2002), para 5.52, 5.55.

74 JA 397/266: September 1999. David Flory, Dr Ruth Hussey, Wendy Jones. A financial review of the Public Health Laboratory Service, pp. 32, 35.

75 JA 397/266: 06 October 1999. Memo. From Peter Lemmey to Alice Perkins. PHLS – Flory report on financial management.

76 JA 397/266: nd. Financial review of the Public Health Laboratory Service. Response of the PHLS board, p10.

77 JA 397/263 PHLS: board meetings—papers + minutes: 05 May 2000. Memo. From Andy Smith PH7. To Dr Troop, DCMO. PHLS Board meeting: 4 May 2000.

78 JA 397/263: PHLSB 00/23 04 May 2000. Minutes of the meeting of the Board, p8.

79 JA 397/267 Implementation of the Flory report: 08 March 2000. Memo. From John Bywater PH7C. To Permanent secretary. Meeting with Sir Leslie Turnberg.

80 JA 397/263: 14 January 2000. Memo. From John Bywater PH7. To Dr Troop DCMO, Dr Leese PH6, Mr Smith PH7, Mr Stopes-Roe RMF1, Mr Brown PH6, Miss Farr RMF1. PHLS corporate and business plans.

81 Department of Health, *Getting Ahead of the Curve*; Kirchhelle, *Emergencies & Omissions*, 26-27.

82 Department of Health, *Getting Ahead of the Curve*, 133.

83
*Ibid*., 105, 112, 114, 139.

84 JA 397/263: PHLSB 00/26 04 May 2000. Minutes of the Finance and General Purposes Committee, p2.

85 JA 397/267: 08 March 2000. Memo. From John Bywater PH7C. To Permanent Secretary. Meeting with Sir Leslie Turnberg, para 12.

86 JA 397/250 PHLSB: board meeting papers: PHLSB 02/50 18 April 2002. Minutes of the meeting of the Board, Annex. Draft letter to secretary of state.

87 David Dance, ‘Memorandum’. Select Committee on Science and Technology Written Evidence (2002) https://publications.parliament.uk/pa/ld200203/ldselect/ldsctech/23/23w19.htm.

88 T. Hugh Pennington, ‘Memorandum’, Select Committee on Science and Technology Written Evidence (2002) https://publications.parliament.uk/pa/ld200203/ldselect/ldsctech/23/23w46.htm.

89 Academy of Medical Sciences, ‘Memorandum’, Select Committee on Science and Technology Written Evidence (2002) https://publications.parliament.uk/pa/ld200203/ldselect/ldsctech/23-ii/2102902.htm; Keith A. V. Cartwright, ‘Memorandum’, Select Committee on Science and Technology Written Evidence (2002) https://publications.parliament.uk/pa/ld200203/ldselect/ldsctech/23/23w50.htm; Nastasha S. Crowcroft, ‘Memorandum’, Select Committee on Science and Technology Written Evidence (2002) https://publications.parliament.uk/pa/ld200203/ldselect/ldsctech/23/23w18.htm; Dance, ‘Memorandum’; Faculty of Public Health Medicine, ‘Memorandum’, Select Committee on Science and Technology Written Evidence (2002) https://publications.parliament.uk/pa/ld200203/ldselect/ldsctech/23/23w22.htm; Sheffield City Council, the Health Protection Services, ‘Memorandum’, Select Committee on Science and Technology Written Evidence (2002) https://publications.parliament.uk/pa/ld200203/ldselect/ldsctech/23/23w59.htm; Michael O’Brien, ‘Health Protection: The Final Straight?’ *Public Health*, 2002, 116, 184–85.

90 Stephanie Snow, Anthony Kessel, and Emmeline Buckley, *The History of the Health Protection Agency 2003–2013* (London: Public Health England, 2013), 25; Kirchhelle, *Emergencies & Omissions*, 24.

91 Michael O’Brien, ‘Memorandum’, Select Committee on Science and Technology Written Evidence (2002) https://publications.parliament.uk/pa/ld200203/ldselect/ldsctech/23/23w45.htm.

92 Castellani, *Rise of Managerial Bureaucracy*, 155, 172; Lowe and Pemberton, *Official History of the British Civil Service*, 3.

93 Lowe and Pemberton, *Official History of the British Civil Service*, 211.

94 Alex Vass, ‘New Agency Created to Fight Infectious Diseases’, *BMJ*, 2002, 324, 131.

95 Snow, Kessel, and Buckley, *History of the HPA*, 28.

96
*Ibid*., 31, 54, 55.

97 Public Health Laboratory Service Board, *Account 2002-2003* (London: House of Commons, 2004).

98 Kirchhelle, *Emergencies & Omissions*, 29; Liam Donaldson, *The Chief Medical Officer on the State of Public Health: Annual Report 2005*. (London: Department of Health, 2006), 40; Liam Donaldson, *2006 Annual Report of the Chief Medical Officer on the State of Public Health* (London: Department of Health, 2007), 12.

99 Snow, Kessel and Buckley, *History of the HPA*, 53.

100 Department of Health, *The Government’s Response to the House of Commons Health Committee’s First Report on the Role of the Private Sector in the NHS* (London: Department of Health, 2002).

101 Department of Health, *The New NHS: Modern, Dependable* (London: The Stationery Office 1997).

102 Donaldson (Sir Liam) archive, Newcastle University LD-03-03-09 Memo. From Prof. Liam Donaldson CMO. To secretary of state. Reorienting the NHS—some ideas: 30 October 2001. Donaldson also suggested running the NHS outside the DH.

103 Department of Health, *The New NHS: Modern, Dependable*, 50; O’Brien, ‘Memorandum’.

104 JA 397/250: PHLSB 99/ 14 January 1999. Minutes of the meeting of the Board.

105 JA 397/266: September 1999. David Flory, Dr Ruth Hussey, Wendy Jones. A financial review of the Public Health Laboratory Service, pp. 11–13.

106 JA 397/135: 18 October 1994. Memo. From Dr E Tebbs HP(M)1. To JG Brown HEF(A)4. PHLS strategy review.

107 JA 397/41: 25 March 1998. Note of a DH/PHLS meeting on 25 March 1998, para 11.

108 Cartwright, ‘Memorandum’; Dance, ‘Memorandum’, Sheffield City Council, ‘Memorandum’.

109 JA 397/135: 21 November 1994. Letter. From Peter Kendall. To Diana Walford. Strategic review of the PHLS.

110 JA 397/92/1 PHLS review 1991: 22 August 1991. Memo. Dr Ailsa Wright to Mr Brown. PHLS review.

111 Snow, Kessel, and Buckley, *History of the HPA*, 31, 33.

112 Michael O’Brien *et al*., ‘Tempting Fate: Control of Communicable Disease in England’. *BMJ*, 1993, 306, 1461–64.

113 David Satcher, ‘Emerging Infections: Getting Ahead of the Curve’. *Emerging Infectious Diseases*, 1995, 1, 1–6.

114 Health Protection Agency, *Annual Report and Accounts 2004* (London: House of Commons, 2004), 3.

115 Health and Social Care (Community Health and Standards) Act 2003, section 190; The Health and Social Care (Community Health and Standards) Act 2003 (Commencement) (No. 6) Order 2005, Article 2(1)(a).

116 Brian Duerden, ‘Twenty-First-Century Medical Microbiology Services in the UK’, *Nature Reviews Microbiology*, 2005, 3, 979–83.

117 PHLS, *Account 2002-2003*, 3.

118 Lord Carter of Coles, *Report of the Review of NHS Pathology Services in England* (Department of Health, 2006), 43.

119 David Dance, and Brian Duerden, *The Health Protection Functions of NHS Diagnostic Microbiology Laboratories* (London: Health Protection Agency, 2006).

120 Dance, ‘Memorandum’.

121 Christine McCartney, ‘Regional Microbiology Network’ *British Journal of Infection Control*, 2008, 9, 28–9.

122 Carter, *Review of NHS Pathology Services*, 134; Lord Carter of Coles, *Report of the Second Phase of the Review of NHS Pathology Services in England* (London: Department of Health, 2008), 23.

123 Public Health England, *Annual Report and Accounts 2014/15* (London: Public Health England, 2015). https://www.gov.uk/government/publications/public-health-england-annual-report-and-accounts-2014-to-2015.

124 Leslie Turnberg, ‘Lessons from the Past for New Health Agency’ (*The Times*, 19 August 2020). https://www.thetimes.co.uk/article/times-letters-lessons-from-the-past-for-new-health-agency-0wxjgjg2d.

125 Brian Duerden *et al*., *The Laboratory Response to the Covid-19 Pandemic* (n.d.).

126 Snow, Kessel and Buckley, *History of the HPA*, 50, 72.

127 Faculty of Public Health Medicine, ‘Memorandum’.

128 Valerie Bevan, ‘It’s Goodbye PHE, and Hello National Institute for Health Protection’, *Pathology in Practice*, 2020, 21–23.

129 Kirchhelle, ‘Giants on Clay Feet’, 44–5.

130 Peter Roderick, Allyson M. Pollock, and Alison Macfarlane. ‘Getting Back on Track: Control of Covid-19 Outbreaks in the Community’, *BMJ*, 2020, 369, m2484.

131 Public Health England. ‘PHE Novel Coronavirus Diagnostic Test Rolled out across UK’. GOV.UK (2020). https://www.gov.uk/government/news/phe-novel-coronavirus-diagnostic-test-rolled-out-across-uk.

## Notes

James Lancaster is an associate researcher at Newcastle University.

Peter Roderick is a principal research associate at Newcastle University. He was called to the Bar by Gray’s Inn in 1982. He worked in private practice and as a public interest environmental lawyer. As an academic researcher, he specialises in health and pharmaceutical law and human rights law.

Allyson Pollock trained in medicine and is a clinical professor of public health at Newcastle University. Her research interests include access to medicines and appropriate medicines use, pharmaceutical regulation and regulatory science, the epidemiology of child and sports injury, public-private partnerships and health systems, and long-term care.

